# Evaluating the Usefulness of a Novel ^10^B-Carrier Conjugated With Cyclic RGD Peptide in Boron Neutron Capture Therapy

**DOI:** 10.4021/wjon477w

**Published:** 2012-07-05

**Authors:** Shin-ichiro Masunaga, Sadaaki Kimura, Tomohiro Harada, Kensuke Okuda, Yoshinori Sakurai, Hiroki Tanaka, Minoru Suzuki, Natsuko Kondo, Akira Maruhashi, Hideko Nagasawa, Koji Ono

**Affiliations:** a Particle Radiation Oncology Research Center, Research Reactor Institute, Kyoto University, 2-1010, Asashiro-nichi, Kumatori-cho, Sennan-gun, Osaka 590-0494, Japan; b Laboratory of Medicinal and Pharmaceutical Chemistry, Gifu Pharmaceutical University, 1-25-4 Daigaku-nishi, Gifu 501-1196, Japan; c Radiation Medical Physics, Research Reactor Institute, Kyoto University, 2-1010, Asashiro-nichi, Kumatori-cho, Sennan-gun, Osaka 590-0494, Japan

**Keywords:** Boron neutron capture therapy, Integrin, Quiescent cell, Mercaptododecaborate-^10^B, Cyclodextrin

## Abstract

**Background:**

To evaluate the usefulness of a novel ^10^B-carrier conjugated with an integrin-binding cyclic RGD peptide (GPU-201) in boron neutron capture therapy (BNCT).

**Methods:**

GPU-201 was synthesized from integrin-binding Arg-Gly-Asp (RGD) consensus sequence of matrix proteins and a ^10^B cluster 1, 2-dicarba-*closo*-dodecaborane-^10^B. Mercaptododecaborate-^10^B (BSH) dissolved in physiological saline and BSH and GPU-201 dissolved with cyclodextrin (CD) as a solubilizing and dispersing agent were intraperitoneally administered to SCC VII tumor-bearing mice. Then, the ^10^B concentrations in the tumors and normal tissues were measured by γ-ray spectrometry. Meanwhile, tumor-bearing mice were continuously given 5-bromo-2’-deoxyuridine (BrdU) to label all proliferating (P) cells in the tumors, then treated with GPU-201, BSH-CD, or BSH. Immediately after reactor neutron beam or γ-ray irradiation, during which intratumor ^10^B concentrations were kept at levels similar to each other, cells from some tumors were isolated and incubated with a cytokinesis blocker. The responses of the Q and total (= P + Q) cell populations were assessed based on the frequency of micronuclei using immunofluorescence staining for BrdU.

**Results:**

The ^10^B from BSH was washed away rapidly in all these tissues and the retention of ^10^B from BSH-CD and GPU-201 was similar except in blood where the ^10^B concentration from GPU-201 was higher for longer. GPU-201 showed a significantly stronger radio-sensitizing effect under neutron beam irradiation on both total and Q cell populations than any other ^10^B-carrier.

**Conclusion:**

A novel ^10^B-carrier conjugated with an integrin-binding RGD peptide (GPU-201) that sensitized tumor cells more markedly than conventional ^10^B-carriers may be a promising candidate for use in BNCT. However, its toxicity needs to be tested further.

## Introduction

Boron neutron capture therapy (BNCT) is, in principle, very promising modality for cancer treatment provided that a sufficient amount of ^10^B can be accumulated in the target tumor and a sufficient number of very-low-energy thermal neutrons can be delivered there. BNCT is based on the neutron capture and fission reaction of non-radioactive ^10^B, (^10^B (n, α)^7^Li). This results in selective boron-localized cell killing because the particles generated in this reaction have a short range, less than 14 µm (the approximate diameter of 1-2 cells), and carry high linear energy transfer [1]. Therefore, tumor-selective delivery of a sufficient amount of ^10^B atoms is essential to the success of BNCT.

Disodium mercaptododecaborate (BSH, Na_2_^10^B_12_H_11_SH, MW = 211.3) and *p*-boronophenylalanine (BPA, C_9_H_12_^10^BNO_4**,**_ MW = 208.2) are ^10^B-carriers for clinical investigation, and currently undergoing clinical trials for BNCT [1]. Neither boron carriers, however, is ideal for delivering a therapeutic amount of ^10^B throughout the target tumor because they both tend to be washed out immediately after injection [2]. In recent years, great progress has been made towards targeting integrins in cancer treatment. Integrins are a superfamily of heterodimeric transmembrane receptors comprising 19 α-subunits and 9 β-subunits which participate in cell-cell and cell-matrix interactions. Integrins regulate a diverse array of cellular functions related to progression, angiogenesis, and metastasis in the tumor microenvironment [3]. Among the integrin family, integrin αvβ3 is the most attractive target for anti-tumor drug delivery and tumor imaging due to its specific expression on proliferating endothelial cells and tumor cells of various origins [3].

Therefore, to enhance the tumor-specific uptake of ^10^B atoms, we developed new tumor-selective ^10^B-carriers bearing an integrin-binding Arg-Gly-Asp (RGD) sequence of matrix proteins for binding integrins at the cell surface. We carried out the design and synthesis of novel ^10^B-carriers conjugated with cyclic RGD peptides directed at integrin αvβ3, using two icosahedral ^10^B clusters, that is, BSH or 1, 2-dicarba-closo-dodecaborane-^10^B (*o*-carborane) [4]. An *in vitro* cell adhesion assay on αvβ3-positive U87MG and SCC VII cells demonstrated the high affinity of the conjugates for integrin αvβ3. Biodistribution experiments using SCC VII-bearing mice indicated that one of the newly synthesized ^10^B-carriers GPU-201 showed good tumor uptake and a significantly longer retention in tumors than the other newly synthesized ^10^B-carriers or BSH [4].

Based on these characteristics, we evaluated the usefulness of GPU-201 as a ^10^B-carrier for BNCT in terms of ^10^B biodistribution analyses and radio-sensitization effects with thermal neutrons or γ-rays on both total (= proliferating (P) + quiescent (Q)) and Q cell populations in solid tumors, including tumor growth delay assay.

## Materials and Methods

### Mice and tumors

SCC VII squamous cell carcinomas (Department of Radiology, Kyoto University) derived from C3H/He mice were maintained *in vitro* in Eagle’s minimum essential medium supplemented with 12.5% fetal bovine serum. Cells were collected from exponentially growing cultures, and 1.0 x10^5^ cells of each tumor were inoculated subcutaneously into the left hind legs of 8- to 11-week-old syngeneic female C3H/He mice (Japan Animal Co., Ltd., Osaka, Japan). Fourteen days after the inoculation, each tumor had reached approximately 1 cm in diameter. At treatment, the body weight of the tumor-bearing mice was 22.1 ± 2.3 (mean ± standard deviation) g. Mice were handled according to the Recommendations for Handling of Laboratory Animals for Biomedical Research, compiled by the Committee on Safety and Ethical Handling Regulations for Laboratory Animal Experiments, Kyoto University. All experimental procedures mentioned here were in accordance with institutional guidelines for the care and use of laboratory animals in research.

### Compounds

**Figure 1:**
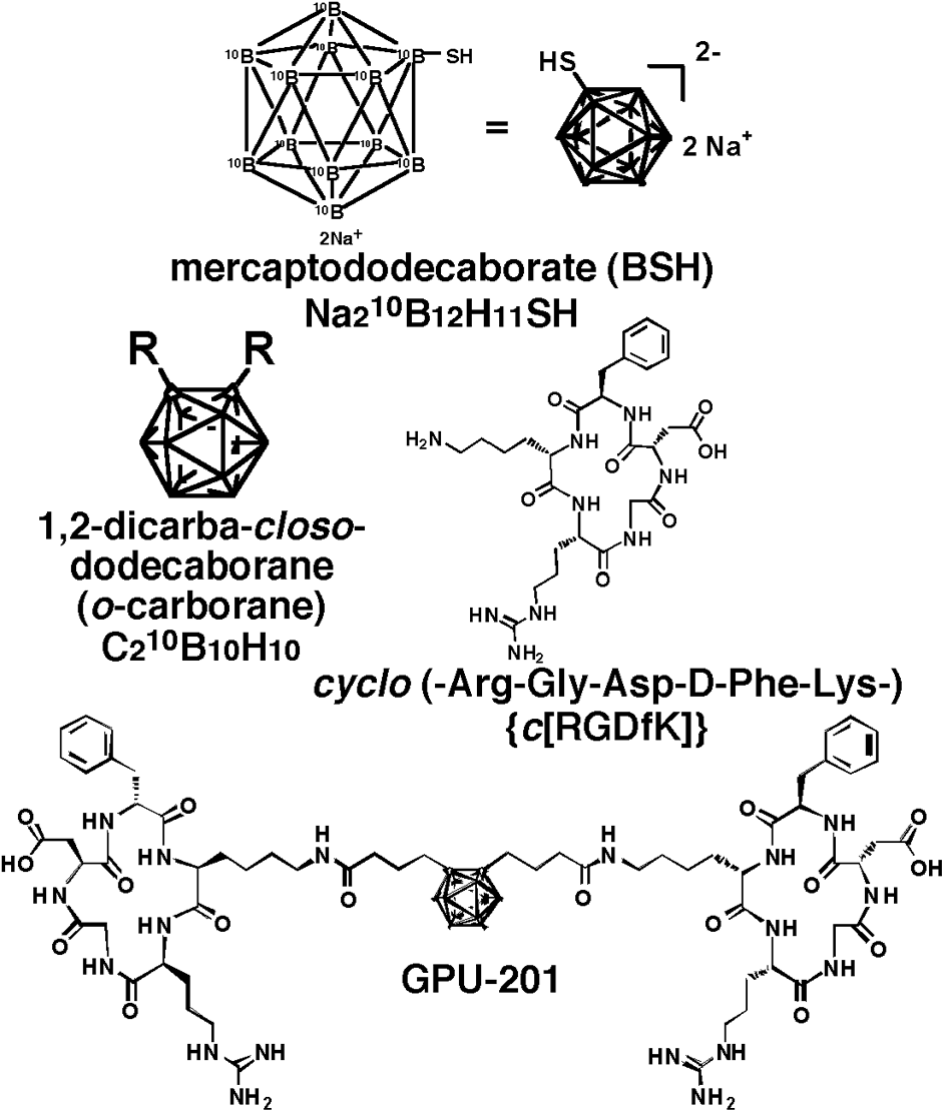
Chemical structure of disodium mercaptododecaborate-^10^B (BSH), 1, 2-dicarba-closo-dodecaborane (o-carborane), cyclo(-Arg-Gly-Asp-D-Phe-Lys-) {c(RGDfK)} and GPU-201.

The cyclo (-Arg-Gly-Asp-D-Phe-Lys-) {*c*(RGDfK)} (Fig. 1) was synthesized as a tumor-targeting moiety by the Fmoc solid-phase method and conjugated to a ^10^B cluster, 1, 2-dicarba-closo-dodecaborane (*o*-carborane) (Fig. 1), through an alkyl amide linker chain. *o*-Carborane has two adjacent carbon atoms thereby enabling construction of a different type of RGD dimer by the attachment of c(RGDfK) to each carbon atom via a linker. GPU-201 (C_64_H_102_^10^B_10_N_18_O_16_, MW = 1479.74) (Fig. 1) has a symmetric RGD-dimer. Details of its synthesis were described elsewhere [4].

BSH (Fig. 1) was purchased from Kat Chem Ltd. (Czech Republic), and prepared freshly by dissolving in physiological saline and injected intraperitoneally in a volume of 0.02 mL/g mouse body weight. In accordance with our previous study [5], BSH showed no overt toxicity at a dose of less than 500 mg/kg. Based on the certificate of analysis and Material Safety Data Sheet provided by the manufacturer, it was not contaminated with the borocapatate dimer (BSSB, (^10^B_24_H_22_S_2_)^4-^).

### Biodistribution experiment

BSH dissolved in physiological (0.9%) saline only and BSH and GPU-201 dissolved in physiological saline containing 10% 2-hydroxypropyl-β-cyclodextrin (HP-β-CD) as a solubilizing and dispersing agent were administered to the tumor-bearing mice intraperitoneally (0.75 µmole/g body weight of mice). At various time points after the administration, mice were sacrificed and tumors were excised. Additionally, brain, blood, liver, muscle, and skin samples were also collected. The blood samples were collected through heart puncture. The ^10^B concentrations in these tissues were measured by prompt γ-ray spectrometry using a thermal neutron guide tube installed at the Kyoto University Reactor (KUR) [6].

### Labeling with 5-bromo-2’-deoxyuridine (BrdU)

Nine days after the tumor cell inoculation, mini-osmotic pumps (Durect Corporation, Cupertino, CA, USA) containing BrdU dissolved in physiological saline (200 - 250 mg/mL) were implanted subcutaneously, to label all P cells, for 5 days. Administration of BrdU did not change the tumor growth rate. The tumors were 1 cm in diameter on treatment. The labeling index after continuous labeling with BrdU was 55.3 ± 4.5 (mean ± standard deviation) %, and reached a plateau level at these stages. Therefore, we regarded tumor cells not incorporating BrdU after continuous labeling as Q cells.

### Irradiation

To obtain similar intratumor ^10^B concentrations during exposure to neutrons or γ-rays, irradiation was started from 60 min and 30 min after the intraperitoneal administration of BSH or GPU-201 dissolved in physiological saline containing HP-β-CD at a dose of 0.75 µmole/g and BSH dissolved in physiological saline at a dose of 1.2 µmole/g, respectively.

The tumor-bearing mice were irradiated with a reactor neutron beam at a power of 5 MW at KUR. For irradiation of the tumors implanted into the left hind legs of mice, a device made of acrylic resin and holding 12 mice was used, and the tumor-bearing mice were irradiated with reactor thermal neutron beams or γ-rays after being fixed in position with adhesive tape. A lithium fluoride (LiF) thermoplastic shield was employed to avoid irradiating other parts of the body. Thermal neutron irradiation was performed using a reactor neutron beam with a cadmium (Cd) ratio of 160. The neutron fluence was measured from the radioactivation of gold foil at both the front and back of the tumors. The tumors were small and located just beneath the surface. Therefore, neutron fluence was assumed to decrease linearly from the front to back of the tumors. Thus, we used the average neutron fluence determined from the values measured at the front and back. Contaminating γ-ray, including secondary γ -ray, doses were measured with thermoluminescence dosimeter (TLD) powder at the back of the tumors and Teflon tubes. The TLD used was Beryllium Oxide (BeO) enclosed in a quartz glass capsule. BeO itself has some sensitivity to thermal neutrons. The thermal neutron fluence of 8 x 10^12^ cm^-^^2^ is equal to a dose of approximately 1 cGy of γ-rays. We usually use a TLD together with gold activation foil to correct neutron-sensitivity. Details were described previously [7]. For the estimation of neutron energy spectra, eight kinds of activation foil and 14 kinds of nuclear reaction were used. The absorbed dose was calculated using the flux-to-dose conversion factor [8]. The tumors contained (in weight percentage terms), H (10.7%), C (12.1%), N (2%), O (71.4 %), and others (3.8 %) [9]. The average neutron flux (n.cm^-2^·s^-1^) and Kerma rate (cGy·h^-1^) of the beam were 2.0 x10^9^ and 96 for the thermal neutron range (≤ 0.6 eV), 2.8 x 10^7^ and 1.03 for the epithermal neutron range (0.6 eV - 10 keV), and 6.6 x 10^6^ and 28.4 for the fast neutron range (≥ 10 keV), respectively. The Kerma rate for boron dose per Φ n·cm^-2^·s^-1^ of thermal neutron flux for 1 µg·g^-1^ of ^10^B was 2.67 x 10^-8^ Φ cGy·h^-1^. The contaminating γ -ray dose rate was 120 cGy·h^-1^. γ-Ray irradiation was performed with a cobalt-60 γ-ray irradiator at a dose rate of approximately 2.0 Gy.min^-1^.

Each irradiation group also included mice that were not pretreated with BrdU.

### Immunofluorescence staining of BrdU-labeled cells and micronucleus (MN) assay

Right after the *in vivo* irradiation of the implanted tumors, tumors were excised from the mice given BrdU, minced, and trypsinized (0.05% trypsin and 0.02% ethylenediamine-tetraacetic acid (EDTA) in phosphate-buffered saline (PBS), 37 °C, 15 min). Tumor cell suspensions thus obtained were incubated for 72 h in tissue culture dishes containing complete medium and 1.0 µg·ml^-1^ of cytochalasin-B to inhibit cytokinesis while allowing nuclear division, and the cultures were then trypsinized and cell suspensions were fixed. After the centrifugation of fixed cell suspensions, the cell pellet was resuspended with cold Carnoy’s fixative (ethanol:acetic acid = 3:1 in volume). The suspension was then placed on a glass microscope slide and the sample was dried at room temperature. The slides were treated with 2 M hydrochloric acid for 60 min at room temperature to dissociate the histones and partially denature the DNA. The slides were then immersed in borax-borate buffer (pH 8.5) to neutralize the acid. BrdU-labeled tumor cells were detected by indirect immunofluorescence staining using monoclonal anti-BrdU antibody (Becton Dickinson, San Jose, CA, USA) and fluorescein isothiocyanate (FITC)-conjugated antimouse IgG antibody (Sigma, St. Louis, MO, USA). To observe the double staining of tumor cells with green-emitting FITC and red-emitting propidium iodide (PI), cells on the slides were treated with PI (2 µg/mL in PBS) and monitored under a fluorescence microscope.

When cell division is disrupted, or the chromosomes are broken or damaged by chemicals or radiation, then the distribution of genetic material between the two daughter nuclei during cell division is affected and pieces or entire chromosomes fail to be included in either of the two daughter nuclei. The genetic material that is not incorporated into a new nucleus forms its "micronucleus". Thus, the frequency of micronucleus formation reflects the genotoxicity of a chemical compound and radiation very well. The MN frequency in cells not labeled with BrdU could be examined by counting the micronuclei in the binuclear cells that showed only red fluorescence. The MN frequency was defined as the ratio of the number of micronuclei in the binuclear cells to the total number of binuclear cells observed. The ratios obtained in tumors not pretreated with BrdU indicated the MN frequency at all phases in the total (P + Q) tumor cell population. More than 400 binuclear cells were counted to determine the MN frequency [10].

### Clonogenic cell survival assay

The clonogenic cell survival assay was also performed in the mice given no BrdU using an *in vivo*-*in vitro* assay method. Tumors were disaggregated by stirring for 20 min at 37 °C in PBS containing 0.05% trypsin and 0.02% EDTA. The cell yield was (4.5 ± 0.9) x 10^7^ g^-1^ tumor weight. Appropriate numbers of viable tumor cells from the single cell suspension were plated on 60 or 100 mm tissue culture dishes, and, 12 days later, colonies were fixed with ethanol, stained with Giemsa, and counted. For the tumors that received no irradiation, the plating efficiencies for the total tumor cell populations and the MN frequencies for the total and Q cell populations are shown in Table 1.

**Table 1:** Plating Efficiencies and Micronucleus Frequencies at 0 Gy

No	^10^B-carrier	BSH*	BSH-CD#	GPU-201#
Plating efficiency (%) Total cell population	70.7 ± 8.8**	64.0 ± 5.0	14.9 ± 1.7	14.3 ± 1.6
Micronucleus frequency Total cell population	0.027 ± 0.005	0.034 ± 0.004	0.067 ± 0.006	0.070 ± 0.007
Quiescent cells	0.053 ± 0.004	0.057 ± 0.005	0.090 ± 0.010	0.100 ± 0.012

*: Sodium borocaptate-^10^B dissolved in physiological (0.9%) saline; #: BSH and GPU-201 dissolved in physiological (0.9%) saline containing 10% 2-hydroxypropyl-β-cyclodextrin (HP-β-CD); **: Mean ± standard deviation (n = 9).

### Growth of SCC VII tumors

After neutron beam irradiation at an absorbed dose of 0 or 1.825 Gy with or without a ^10^B-carrier on the 14th day after inoculation, the size of the tumors implanted in the left hind legs of some tumor-bearing mice was checked 2 - 3 times a week for about 4 weeks. Tumor volume was calculated using the formula: V = π/6 x *a* x *b*^2^, where *a* and *b* are respectively the longest and shortest diameters of the tumor measured with calipers.

Three mice were used to assess each set of conditions and each experiment was repeated twice. To examine the differences between pairs of values, Student’s *t*-test was used when variances of the two groups could be assumed to be equal; otherwise the Welch *t*-test was used. P-values are from two-sided tests.

## Results

Based on the data in Table 1, GPU-201 and BSH-CD (BSH dissolved in physiological saline containing HP-β-CD) treatment induced a significantly lower plating efficiency and higher MN frequency in both the Q and total cellpopulations (P < 0.05) than no drug treatment. BSH (BSH dissolved in physiological saline only without HP-β-CD) treatment caused no significant changes in the plating efficiency or MN frequency of either population compared with no drug treatment.

**Figure 2:**
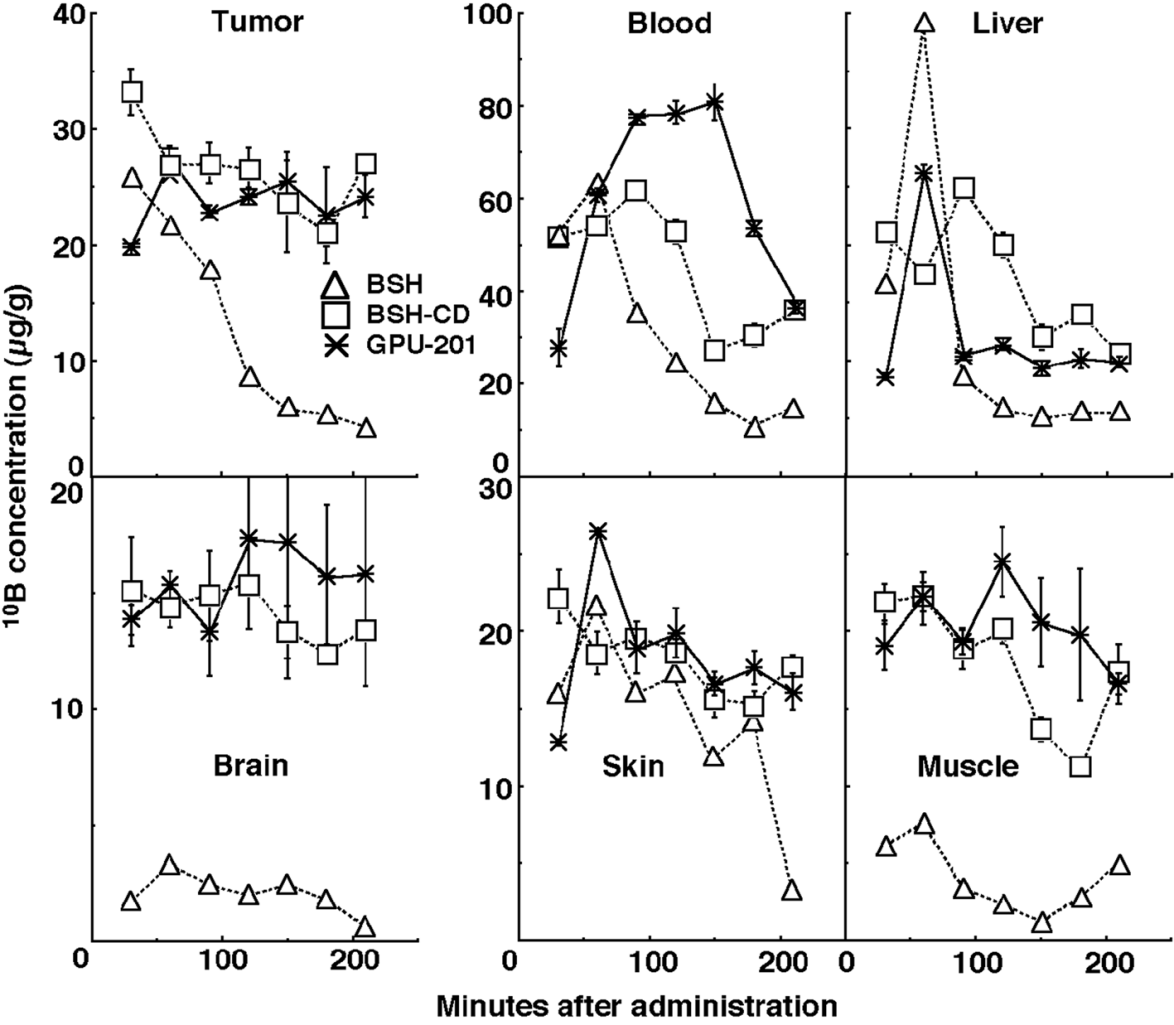
Time course of changes in ^10^B concentrations in the solid tumors, brain, blood collected from heart, liver, muscle, and skin of SCC VII tumor-bearing mice after intraperitoneal administration of each ^10^B-carrier at a dose of 0.75 µmole/g. △: BSH, □: BSH-CD,*: GPU-201.

Figure 2 shows the time course of change in the values of the ^10^B concentration in solid tumors, brain, blood, liver, muscle, and skin. Although the concentrations of ^10^B were actually low in the brain and the concentrations of ^10^B in muscle and skin were a little lower than those in the tumors, the concentrations of ^10^B in blood and liver were higher than those in the tumors. On the whole, ^10^B from BSH in all these tissues was washed away more rapidly than that from any other compound. In contrast, the retention of ^10^B from BSH-CD and GPU-201 was similar except in blood, where the concentration of ^10^B from GPU-201 was higher for longer. The ^10^B concentrations shown here were not thought to be especially toxic [11]. Not as many neutron capture reactions between ^10^B and thermal neutrons are caused in skin as in tumors since epithermal neutron beams are often employed in current BNCT to obtain thermalized epithermal neutrons at the depth of tumors and spare the damage to surface skin.

Based on the findings concerning these ^10^B biodistribution patterns, irradiation was started from 60 min and 30 min after the intraperitoneal administration of BSH-CD or GPU-201 at a dose of 0.75 mole·g^-1^ and BSH at a dose of 1.2 µmole·g^-1^, respectively, because it took approximately 60 min to deliver a sufficient physical dose of radiation to solid tumors with reactor neutron beams. The ^10^B concentrations in tumors for the BSH-CD, GPU-201 and BSH administration groups were 27.5 ± 2.3 µg·g^-1^, 25.5 ± 2.0 µg·g^-1^ and 33.1 ± 4.3 µg·g^-1^, respectively.

**Figure 3:**
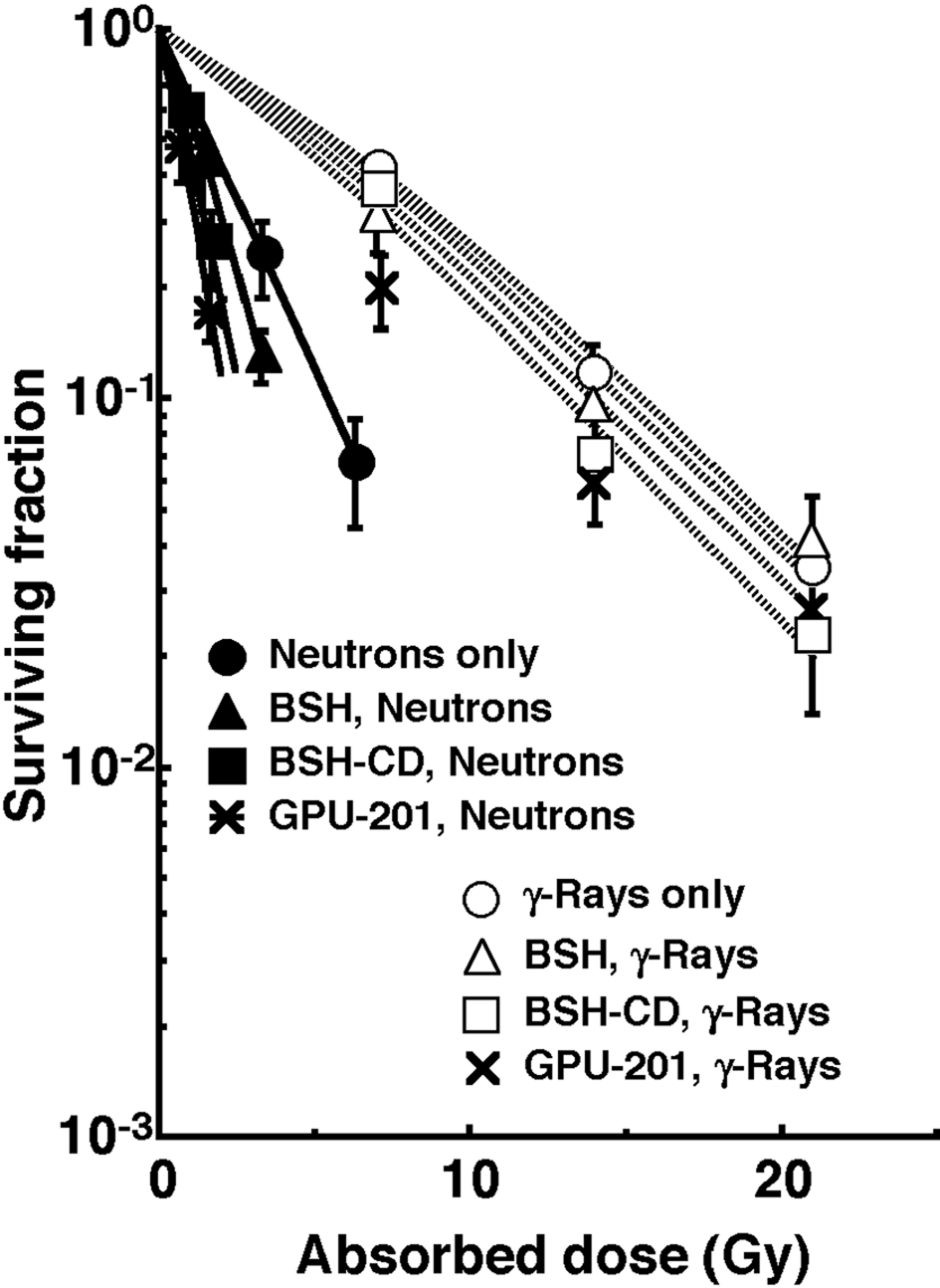
Clonogenic cell survival curves after irradiation in vivo using reactor neutron beams (•, ▲, ■, *) or γ-rays (○, △, □, x) following administration of a ^10^B-carrier (GPU-201, BSH-CD or BSH). •, ○: without a ^10^B-carrier, ▲, △: with BSH, ■, □: with BSH-CD, *, x: with GPU-201. Bars represent standard errors (n = 9).

Figure 3 shows the clonogenic cell survival curves after *in vivo* irradiation using reactor neutron beams or γ-rays following administration of a ^10^B-carrier (GPU-201, BSH-CD or BSH). Overall, neutron beam irradiation induced much greater cytotoxicity in tumor cells than did γ-ray irradiation. Under neutron beam irradiation, the sensitivity of tumor cells was increased with any ^10^B-carrier, however GPU-201 and BSH-CD showed a greater radio-sensitizing effect than BSH. In contrast, no reliable radio-sensitizing effect was obtained with any ^10^B-carrier under γ-ray irradiation.

**Figure 4:**
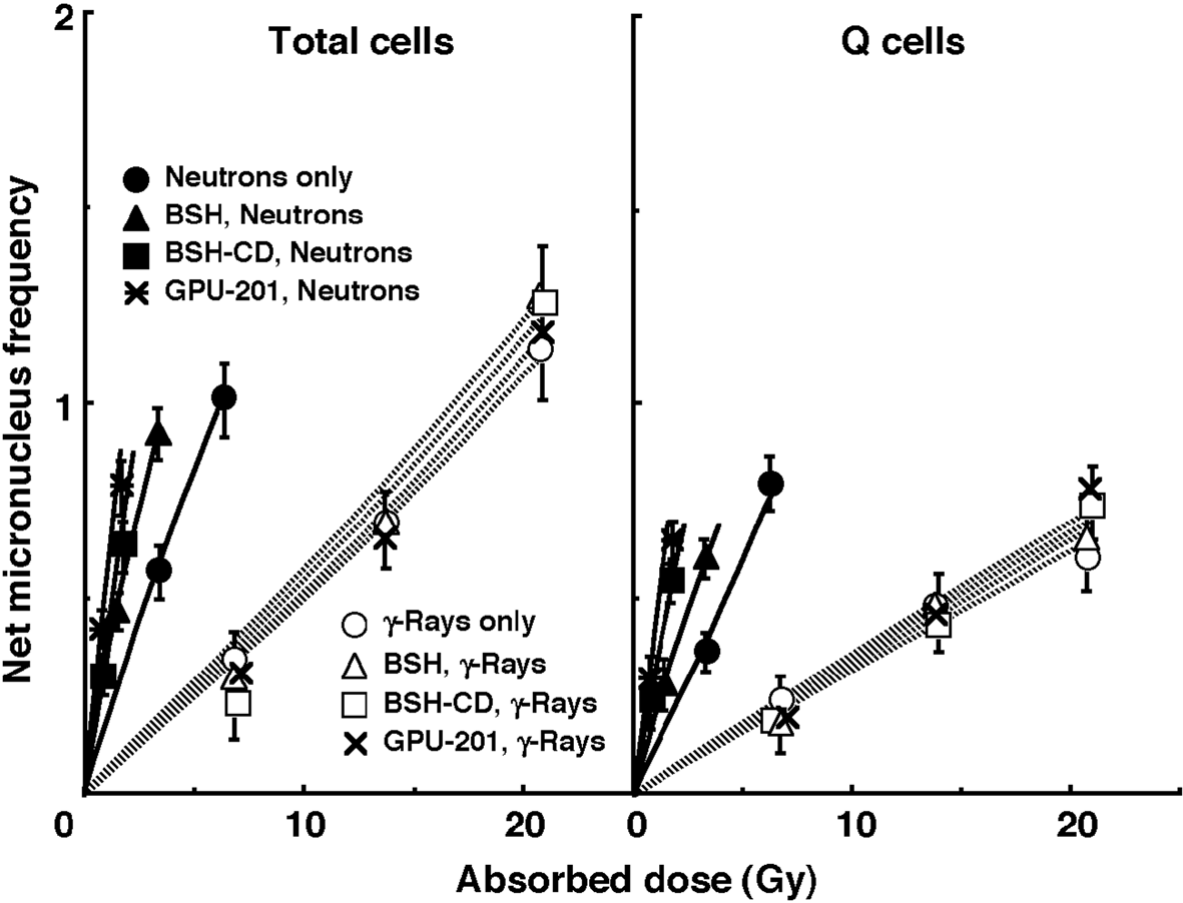
Net micronucleus (MN) frequencies after irradiation in vivo using reactor neutron beams (•, ▲, ■, *) or γ-rays (○, △, □, x) following administration of ^10^B-carrier (GPU-201, BSH-CD or BSH) as a function of the physically absorbed radiation dose in total (left panel) and quiescent (Q, right panel) tumor cell populations. •, ○: without a ^10^B-carrier, ▲, △: with BSH, ■, □: with BSH-CD, *, x: with GPU-201. Bars represent standard errors (n = 9).

The left and right panels of Figure 4 show the net MN frequencies for irradiation *in vivo* as a function of the physically absorbed radiation dose in the total and Q tumor cell populations, respectively. When the ^10^B-carrier was employed, even if no radiation was given, MN frequencies were higher than when no ^10^B-carrier was administered, because of the slight genotoxicity of the drug (Table 1). Therefore, for background correction, we used the net MN frequency to exclude the effects of the genotoxicity of the ^10^B-carrier. The net frequency is the frequency in the irradiated tumors minus the frequency in the nonirradiated tumors. All net MN frequencies were actual values induced under each set of conditions. Namely, they included the values due to contaminating fast neutrons and γ-rays in the neutron beam.

To assess the effect of the ^10^B-carrier on the surviving fractions in the total cell populations and the net MN frequencies in the total and Q cell populations, the enhancement ratio (ER) was calculated using the data given in Figures 3 and 4 (Table 2). Under γ-ray irradiation, there was no significant enhancement with any ^10^B-carrier in both cell populations. Under neutron irradiation, the ER values were significantly larger for the total cells than Q cell populations (P < 0.05) in combination with BSH or BSH-CD, and BSH-CD showed slightly larger ER values than BSH in both populations. Meanwhile, with GPU-201, the values for Q cells were almost equal to those for the total cell population. Further, the ER values were significantly larger for GPU-201 than BSH-CD or BSH, not only in the total tumor cell population but also in Q cells (P < 0.05).

**Table 2:** The Effects* of Drugs on Each Endpoint

	BSH#	BSH-CD**	GPU-201**
Surviving fraction = 0.3 Total cell population			
Neutron Beams	1.4 (1.3-1.5)##	1.7 (1.5-1.9)	2.3 (2.1-2.5)
γ-Rays	1.05 (1.0-1.1)	1.15 (1.0-1.3)	1.25 (1.1-1.4)
Net micronucleus frequency = 0.2 Total cell population			
Neutron Beams	1.8 (1.65-1.95)	2.2 (1.9-2.5)	3.2 (2.9-3.5)
γ-Rays	1.05 (1.0-1.1)	1.1 (1.0-1.2)	1.15 (1.05-1.25)
Quiescent cells			
Neutron Beams	1.5 (1.35-1.65)	1.5 (1.35-1.65)	3.2 (2.9-3.5)
γ-Rays	1.0 (1.0-1.1)	1.05 (1.0-1.1)	1.1 (1.0-1.2)

*:The dose of radiation required to obtain each endpoint without a drug in relation to that required to obtain each endpoint with a drug; #: Sodium borocaptate-^10^B dissolved in physiological (0.9%) saline; **: BSH and GPU-201 dissolved in physiological (0.9%) saline containing 10% 2-hydroxypropyl-β-cyclodextrin (HP-β-CD); ##: Numbers in parentheses are 95% confidence limits, determined using mean values, standard deviations, and the numbers of observations on which the means and standard deviations were based.

To examine the difference in radio-sensitivity between the total and Q cell populations, dose-modifying factors were calculated using the data in Figure 4 (Table 3). Under all γ-ray irradiation conditions, the values were significantly larger than 1.0 (P < 0.05) and almost the same (1.7 - 1.75). In contrast, under neutron irradiation, although all values were significantly larger than 1.0 (P < 0.05), they were significantly smaller for neutrons only and GPU-201 than for BSH or BSH-CD (P < 0.05).

**Table 3:** Dose Ratios* for Quiescent Tumor Cells Relative to the Total Tumor Cell Population

	No ^10^B-carrier	BSH#	BSH-CD**	GPU-201**
Net micronucleus frequency = 0.2				
Neutron Beams	1.25 (1.15-1.35)##	1.7 (1.55-1.85)	1.5 (1.4-1.6)	1.25 (1.15-1.35)
γ-Rays	1.7 (1.55-1.85)	1.75 (1.6-1.9)	1.75 (1.6-1.9)	1.7 (1.55-1.85)

*:The dose of radiation required to obtain each net micronucleus frequency in quiescent tumor cells in relation to that required to obtain each net micronucleus frequency in the total tumor cell population; #: Sodium borocaptate-^10^B dissolved in physiological (0.9%) saline; **: BSH and GPU-201 dissolved in physiological (0.9%) saline containing 10% 2-hydroxypropyl-β-cyclodextrin (HP-β-CD); ##; Numbers in parentheses are 95% confidence limits, determined using mean values, standard deviations, and the numbers of observations on which the means and standard deviations were based.

**Figure 5:**
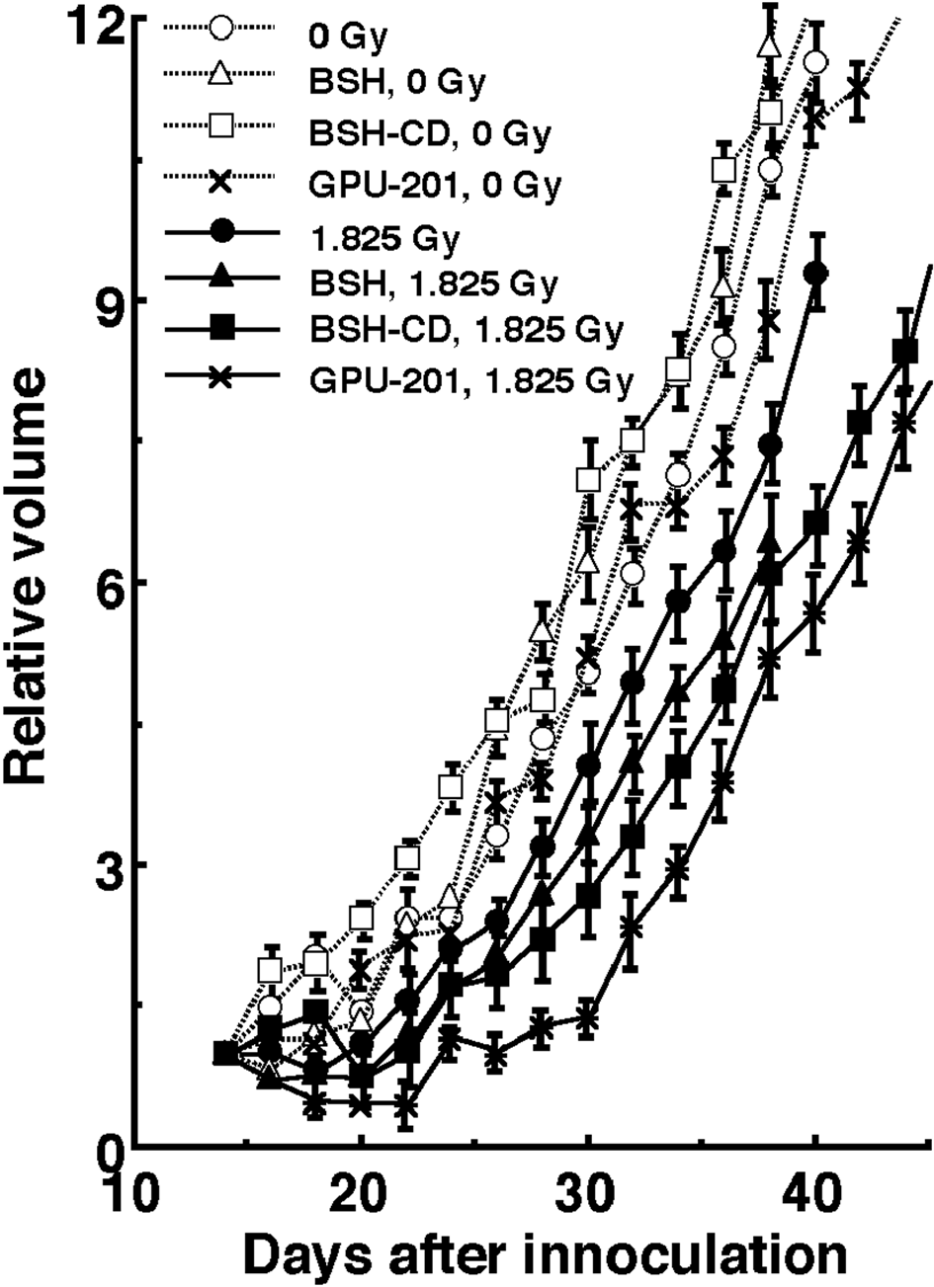
Tumor growth curves for SCC VII tumors with (•, ▲, ■, *) or without (○, △, □, x) neutron beam irradiation following the administration of each ^10^B-carrier. •, ○: without a ^10^B-carrier, ▲, △: with BSH, ■, □: with BSH-CD, *, x: with GPU-201. Bars represent standard errors (n = 9).

Figure 5 shows tumor growth curves after neutron beam irradiation with or without a ^10^B-carrier 14 days after the tumor cell inoculation. Tumor progression with an increase of 3 times the initial size at the time of irradiation can be clearly confirmed in the clinic. Therefore, to evaluate tumor growth, the period required for each tumor to become 3 times as large as it was on day 14 was obtained using the data shown in Figure 5 (Table 4). Without irradiation, with or without a ^10^B-carrier, there was no significant difference in the period. With irradiation at a physically absorbed dose of 1.825 Gy, the period required was significantly prolonged compared with no irradiation (P < 0.05), except for neutrons only without a ^10^B-carrier, and the treatments ranked in the following order; without a ^10^B-carrier < with BSH < with BSH-CD < GPU-201.

**Table 4:** The Period (Days) Required for Each Tumor to Become Three Times as Large as on Day 14 After Tumor Cell Inoculation

	No ^10^B-carrier	BSH#	BSH-CD**	GPU-201**
Without irradiation	11.2 (9.7-12.7)##	10.3 (9.0-11.6)	8.6 (7.7-9.5)	11.1 (9.5-12.7)
With irradiation at 1.825 Gy	13.4 (11.9-14.9)	15.0 (13.5-16.5)	17.0 (15.2-19.8)	20.0 (18.0-22.0)

#: Sodium borocaptate-^10^B dissolved in physiological (0.9%) saline; **: BSH and GPU-201 dissolved in physiological (0.9%) saline containing 10% 2-hydroxypropyl-β-cyclodextrin (HP-β-CD); ##: Numbers in parentheses are 95% confidence limits, determined using mean values, standard deviations, and the numbers of observations on which the means and standard deviations were based.

## Discussion

The results of the ^10^B biodistribution experiments (Fig. 2) revealed that the ^10^B from BSH was quickly washed away in all tissues and that the use of HP-β-CD as a solubilizing and dispersing agent was effective in prolonging the retention of ^10^B in all tissues including the tumors. The use of HP-β-CD in BNCT warrants further study.

Under γ-ray irradiation, no apparent reliable radio-enhancing effect on either the total or Q cell population was observed whichever ^10^B-carrier was employed (Table 2). This is why there was no apparent change in the dose-modifying factor showing the difference in radio-sensitivity between the total and Q cell populations under γ-ray irradiation (Table 3). It follows that all these ^10^B-carriers have the appropriate characteristics as ^10^B-carriers in BNCT in terms of showing no radio-sensitizing effect upon exposure to low energy transfer radiation like γ-rays.

Meanwhile, under reactor neutron irradiation, a significant radio-enhancing effect on the total tumor cell population was observed in the following order; BSH < BSH-CD < GPU-201 (Table 2), irrespective of the reverse order for ^10^B concentrations in irradiated tumors as a whole during neutron beam exposure; GPU-201 < BSH-CD < BSH. Further, the changes in tumor growth as a whole were consistent with and well supported the changes in the radio-sensitivity of the total tumor cell population in cell survival curves and dose-response curves of net MN frequency (Tables 2 and 4). On the other hand, concerning the radio-enhancing effect on Q cells, with the use of BSH or BSH-CD, ER values were similar and significantly lower than in the total cell population (Table 2). In contrast, GPU-201 produced a significantly larger ER than the other ^10^B-carriers for both cell populations, and almost the same ER in Q cells as in the total cell population (Table 2). This means that GPU-201 enhanced the sensitivity of not only the total but also the Q cell population more remarkably than any other ^10^B-carrier (Table 2). Therefore, concerning the difference in radio-sensitivity between the total and Q cell populations, although BSH and BSH-CD produced a significantly greater difference (P < 0.05), GPU-201 induced almost the same difference as neutron irradiation without any ^10^B-carrier (Table 3).

Our previous study concerning the characteristics of intratumor Q cells clarified that the Q cell population in SCC VII solid tumors has a much larger hypoxic fraction than the total cell population [12]. Thus, in this study, it was thought that the response of intratumor hypoxic cells to aerobic irradiation *in vivo* would be represented by the response of intratumor Q cells to some extent. Further, it is difficult to deliver a therapeutic amount of ^10^B from currently used ^10^B-carriers throughout the target tumors, especially into intratumor hypoxic cells with low uptake capacities [1, 2]. The effects on both the total and Q cell populations of neutron irradiation in combination with GPU-201 were significantly greater than those of irradiation in combination with BSH or BSH-CD (P < 0.05), even when the ^10^B concentrations in tumors were kept at similar levels during irradiation. Moreover, whereas BSH or BSH-CD tended to enhance the sensitivity of the total cell population more markedly than that of Q cells, GPU-201 increased the sensitivity of both populations to the same extent. This means that the ^10^B from GPU-201 localized to hypoxia-rich Q cells more easily than that from BSH or BSH-CD. This might be partly because hypoxic tumor cells, to which it is difficult to deliver ^10^B from BSH or BSH-CD, could be killed as the ^10^B from GPU-201 can be distributed to areas of intratumor hypoxia or normoxia.

Integrins bind to a variety of plasma and extracellular matrix proteins containing the conserved RGD amino acid sequence and modulate cell adhesion. They have diverse roles in several biological processes including cell migration during development and wound healing, cell differentiation, and apoptosis. Their activities can also regulate the metastatic and invasive potential of tumor cells. Integrin αvβ3 is highly expressed in osteoclasts where it may play a role in bone resorption, and is also abundant in vascular smooth muscle and endothelial cells, and in some tumor cells, where it is involved in angiogenesis and cell migration [3]. Originally, GPU-201 was designed and synthesized as a tumor-selective ^10^B-carrier from a ^10^B cluster o-carborane conjugated with cyclic RGD peptides directed at integrin αvβ3 through an alkyl amide linker chain. Meanwhile, it was reported that hypoxia induces the recruitment of αvβ3 and αvβ5 integrins to the cellular membrane in glioblastoma cell lines, thereby activating the focal adhesion kinase [13, 14]. Thus, integrin αvβ3 is thought to be an attractive target for overcoming the difficulty in delivering ^10^B into hypoxic cells in solid tumors from a ^10^B-carrier.

Solid tumors, especially human tumors, are thought to contain a high proportion of Q cells [15, 16]. The presence of these cells is probably due, in part, to hypoxia and the depletion of nutrition in the tumor core, which is another consequence of poor vascular supply [15, 16]. It has been reported that Q cells have lower radio-sensitivity than P cells in solid tumors *in vivo* (Table 3) [12, 15, 16]. This means that more Q cells survive anticancer treatment than P cells. Consequently, the control of Q cells has a great impact on the outcome of anticancer therapy. GPU-201 not only showed significantly greater effects on both the total and Q cell populations with neutron irradiation than BSH or BSH-CD, but also increased the sensitivity of Q cells as well as that of the total cell population (Table 2), resulting in almost the same difference in sensitivity between the two populations, as with neutron irradiation only. From the viewpoint of the tumor cell-killing effect as a whole, including intratumor Q cell control, GPU-201 may be promising as a ^10^B-carrier for BNCT because of its potential to sensitize hypoxia-rich Q cell populations.

Finally, according to Table 1, GPU-201 is significantly more cytotoxic to both total and Q cell populations than BSH. Therefore, further study to reduce this toxicity will have to be carried out.
